# Applying the Baveno VII Criteria for Variceal Screening to Reduce Endoscopy Costs and Risks to Patients in an Australian Referral Centre

**DOI:** 10.7759/cureus.110653

**Published:** 2026-06-11

**Authors:** Joel Thio, Matthew Mansoor, Alexander Gluch, Asif Shahzad

**Affiliations:** 1 Gastroenterology and Hepatology, Queensland Health, Brisbane, AUS

**Keywords:** alcohol-related cirrhosis, baveno vii, liver fibrosis, metabolic-associated fatty liver disease (mafld), viral hepatitis b and c

## Abstract

Baveno VII was introduced to help identify patients with compensated advanced chronic liver disease (ACLD) who should undergo screening gastroscopy to detect varices needing treatment (VNT), with a positive predictive value exceeding 90%. We performed a study at our centre to evaluate the impact of implementing this strategy, which has the potential to lower both healthcare costs and the risks associated with invasive procedures.

We carried out a retrospective, single-centre analysis between 2021 and 2024, reviewing screening gastroscopies for oesophageal varices. Data reviewed included gastroscopy findings, transient elastography (TE) results performed within the preceding six months, and platelet count. The underlying cause of liver disease was also recorded. The primary aim was to evaluate the real-world application of the Baveno VII recommendations and estimate their impact on screening endoscopy utilisation and associated healthcare costs.

We reviewed a total of 592 gastroscopies performed for variceal screening. Fifty-four patients were eligible for analysis after exclusion criteria were applied. Twenty-one of 54 patients (38.8%) did not meet the criteria for variceal surveillance. Notably, five out of these 21 patients (23.8%) were found to have VNT. Among the 33 patients who did meet the screening criteria, the positive predictive value for detecting VNT was 48.5% in our cohort. Applying the Baveno VII criteria would have excluded 16 patients who ultimately did not have VNT, potentiating cost savings of 29,120 Australian dollars (AUD).

Application of the Baveno VII criteria in our cohort could have reduced unnecessary screening gastroscopies, resulting in cost savings and reduced procedural risk to patients. Although a small number of patients outside the screening criteria were found to have varices needing treatment, these findings should be interpreted in the context of the study’s retrospective design, single-centre setting, and limited sample size. Nevertheless, this study provides a real-world example of the potential for Baveno VII-guided screening to reduce healthcare utilisation, costs, and patient exposure to invasive procedures.

## Introduction

Screening for oesophageal varices has historically been done routinely on diagnosis of liver cirrhosis to initiate prompt primary prophylaxis for the prevention of acute variceal bleeding (AVB). AVB is a serious complication of liver cirrhosis with mortality rates of about 20% at six weeks [1]. Primary prophylaxis using non-selective beta-blockers (NSBB) or endoscopic variceal banding is used for clinically significant oesophageal varices [2].

Endoscopic procedures do not come without their risks, especially in patients with liver cirrhosis, who are a higher-risk group [3]. Endoscopic variceal banding can cause pain and risk of banding ulcers in patients where this technique is used. Predictive non-invasive testing (NIT) can identify patients with a low likelihood of varices needing treatment (VNT), defined as grade II or higher, or with high-risk stigmata, avoiding the risks of endoscopy in these patients and potentially reducing healthcare costs from reduced invasive procedures.

Baveno VII, introduced in 2022, identified that patients with compensated advanced chronic liver disease (ACLD) with transient elastography (TE) of less than 20 kPa and a platelet count of over 150,000 cells/microL had a low risk of clinically significant portal hypertension (CSPH) with VNT, with a negative predictive value of over 90%, avoiding the need for screening gastroscopy [4]. There is increasing evidence that NSBB should be considered in patients with cirrhosis with CSPH to reduce the risk of hepatic decompensation and to only conduct endoscopic variceal surveillance in patients who are unable to tolerate, or have a contraindication to, NSBB [4]. The current worldwide adherence to these criteria remains largely variable in current clinical practice.

We conducted an analysis of consecutive endoscopic variceal surveillance procedures to assess adherence to these guidelines in our centre, looking at the potential benefits of this in the way of potential invasive procedures avoided for patients and cost savings from avoiding unnecessary screening procedures following Baveno VII.

## Materials and methods

We conducted a single-centre, retrospective analysis of consecutive endoscopic variceal surveillance procedures performed in patients who underwent index gastroscopy for variceal screening over a 48-month period from 1 January 2021 to 31 December 2024. Patient recruitment followed predefined inclusion and exclusion criteria based on the Baveno VII consensus (Table 1). Eligible patients were required to have evidence of ACLD, defined by a liver stiffness measurement on TE of ≥10 kPa, without prior hepatic decompensation. Exclusion criteria included a history of hepatic decompensation, portal vein thrombosis, prior liver transplantation, age <18 years, and obesity defined as body mass index (BMI) >30 kg/m², and patients were additionally excluded if they had not undergone a valid FibroScan within six months preceding their screening gastroscopy. Aetiologies of liver disease included both viral and non-viral causes, including alcohol-related liver disease (ARLD) and metabolic-associated steatotic liver disease (MASLD).

**Table 1:** Inclusion and exclusion criteria MASLD: metabolic associated steatotic liver disease; ARLD: alcohol-related liver disease; BMI: body mass index; ACLD: advanced chronic liver disease

Inclusion Criteria	Exclusion Criteria
Gastroscopy performed between 1 January 2021 and 31 December 2024	History of hepatic decompensation
Index gastroscopy undertaken for the purpose of variceal screening	Presence of portal vein thrombosis
Diagnosed liver disease of viral or non-viral aetiology (e.g., viral hepatitis, MASLD, ARLD)	Pediatric patients (<18 years of age)
Evidence of advanced chronic liver disease (ACLD) with liver stiffness ≥10 kPa within six months of screening gastroscopy	History of liver transplantation
No prior history of hepatic decompensation	Obesity, defined as BMI >30 kg/m²

To ensure accuracy and consistency of TE measurements, patients underwent overnight fasting or fasting for at least four hours before the procedure in accordance with unit protocol. FibroScan assessments were performed using the FibroScan® 502 TOUCH device (Echosens, Paris, France) by one of two senior hepatology clinical nurse consultants credentialled in TE, with measurement reliability assessed using the interquartile range over median (IQR/median), where values ≤10% represented highly reliable measurements and values ≤30% were considered reliable [5]. Platelet counts used for analysis were obtained within six months of screening gastroscopy.

Procedure data were obtained through ProvationMD® (Provation Medical, Inc., Minneapolis, Minnesota, USA), a computerized endoscopic reporting system, including patient demographics, indication for procedure, variceal findings, and endoscopic interventions, and electronic medical records were reviewed to collect corresponding TE and platelet data. Oesophageal varices were classified at index gastroscopy using a standard three-tier system as documented in the endoscopy report. VNT were defined as the presence of grade II or higher oesophageal varices and/or the presence of high-risk stigmata (including red wale signs), in keeping with Baveno VII consensus recommendations.

The primary aim of the study was to assess the real-world application of the Baveno VII recommendations within our cohort and estimate their potential impact on screening endoscopy utilisation and associated healthcare costs, with secondary analyses including evaluation of the diagnostic performance of the Baveno VII criteria, including positive and negative predictive values for the identification of VNT. Descriptive and diagnostic accuracy analyses were performed using IBM® SPSS® Statistics software, version 30.0 (IBM Corp., Armonk, NY, USA), and ethics approval for the study was granted by Metro South Human Research Ethics Committee, Brisbane, Queensland, Australia (Reference: X/2024/QMS/112821; dated 8/10/24).

## Results

**Figure 1:**
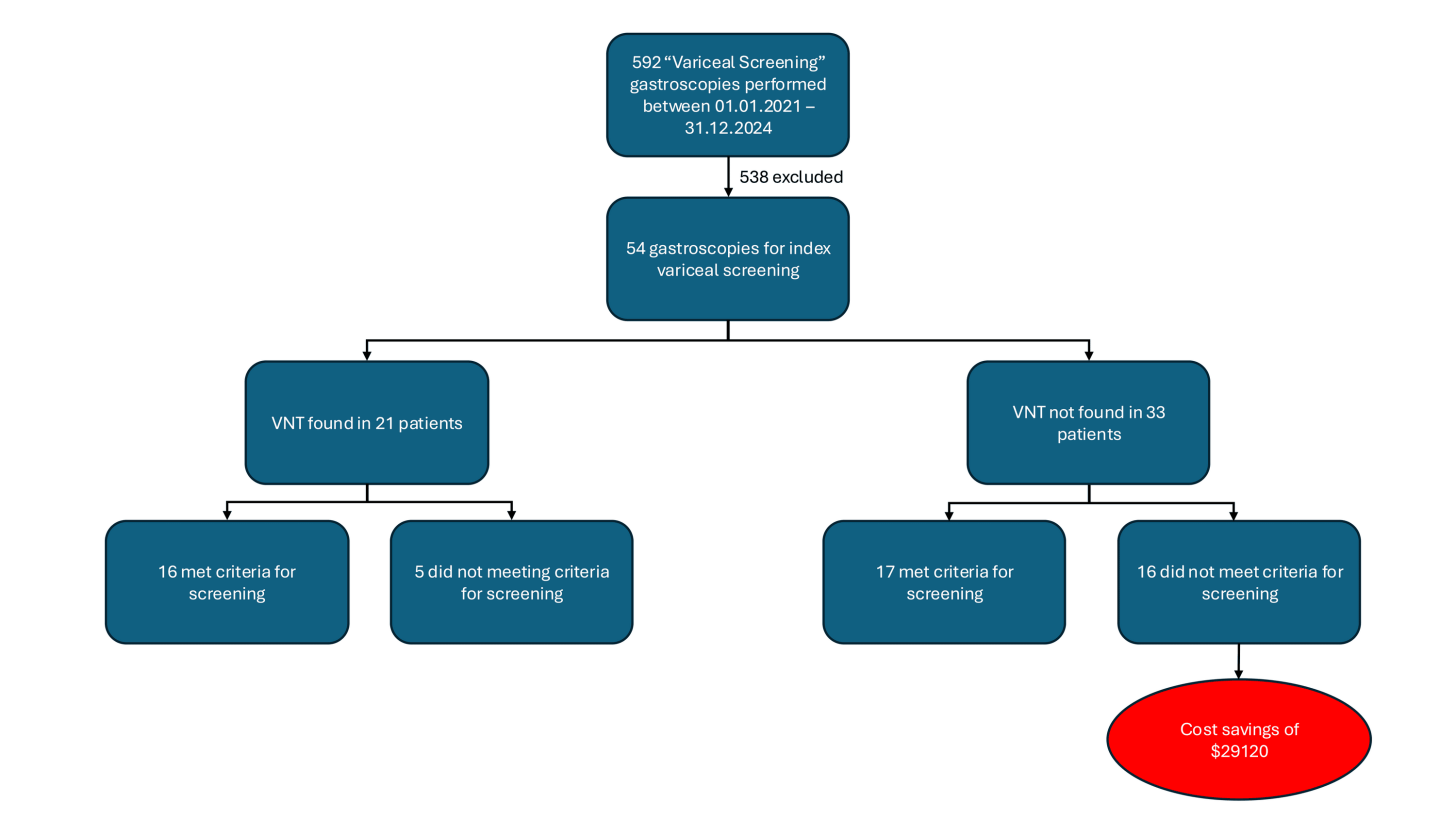
Patients involved in the study VNT: varices needing treatment; Cost savings are in Australian dollars (AUD).

A total of 592 gastroscopies conducted for “variceal screening” were obtained. After excluding gastroscopies done for variceal surveillance and applying the inclusion and exclusion criteria listed in Table 1, 54 (9.1%) patients met the criteria (Figure 1). The mean age was 61 years, with a male predominance of 79.6% (43 patients).

**Figure 2:**
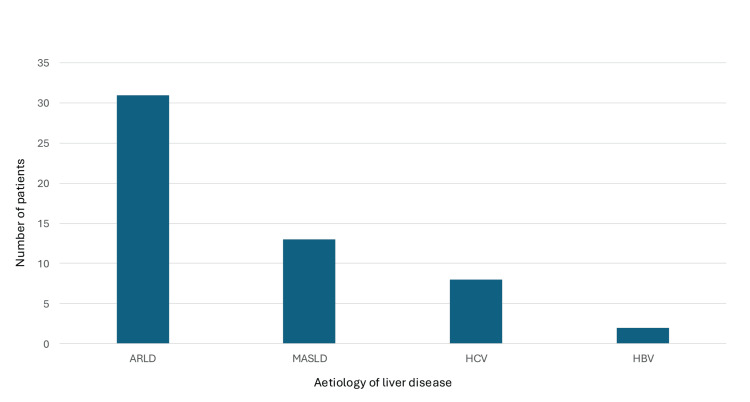
Aetiology of liver disease ARLD: alcohol-related liver disease; MASLD: metabolic-associated steatotic liver disease; HCV: hepatitis C virus; hepatitis B virus

The predominant aetiology of liver disease was ARLD (n=31, 57.4%), followed by MASLD (n=13, 24.1%), hepatitis C (n=8, 14.8%), and the least from hepatitis B (n=2, 3.7%) as shown in Figure 2.

With regards to Fibroscan results, the IQR/median was 10% or under in 90.7% of our study cohort (49 patients). Only five patients (9.3%) had an IQR/median of over 10%, of which the highest reading was 15%.

In this cohort, 33 patients met criteria for undergoing screening gastroscopy to evaluate for VNT. Of these, 16 individuals (48.4%) were found to have VNT on gastroscopy. Data on these 16 patients are shown in Table 2, illustrating the aetiology of liver disease and results of NIT.

**Table 2:** Demographics and details of non-invasive testing of patients who met criteria for endoscopic screening with VNT found ARLD: alcohol-related liver disease; MASLD: metabolic-associated steatotic liver disease; HCV: hepatitis C virus

Age (years)	Gender	Aetiology	Median (kPa)	IQR/Median	Platelet count (x10^3^ cells/microL)
73	Male	ARLD	30.6	10%	191
66	Male	ARLD	29.8	10%	106
41	Male	ARLD	15.8	5%	147
61	Male	ARLD	74	4.4%	186
62	Male	ARLD	30.5	14%	45
76	Female	MASLD	32.1	14%	95
49	Male	ARLD	66	9%	347
81	Male	MASLD	55.1	9%	122
62	Male	HCV (no treatment)	43.5	6%	94
71	Male	MASLD	72.7	4%	75
76	Female	ARLD	27.1	15%	126
36	Male	ARLD	30.4	10%	76
72	Male	MASLD	17.4	4%	149
72	Male	ARLD	17	4%	114
72	Female	MASLD	43.1	7%	158
39	Male	ARLD	30.2	10%	46

Twenty-one patients who did not meet the criteria underwent screening gastroscopy. Among these, five patients (23.8%) were unexpectedly found to have VNT.

Overall, the performance characteristics of Baveno VII in this retrospective cohort yielded a positive predictive value of 48.5% and a negative predictive value (NPV) of 76.2% for the detection of VNT.

In our cohort of eligible patients, 16 of 21 patients who did not meet screening criteria did not have VNT on endoscopy, which represents potential cost savings if Baveno VII were utilised in our centre.

None of the patients in this study cohort who met criteria for CSPH based on Baveno VII were commenced on NSBB prior to screening gastroscopy.

## Discussion

The adherence rate to Baveno VII for screening gastroscopy was 61% in our cohort. Adherence to utilising NSBB in patients who met CSPH criteria, with NIT much lower than expected. Reasons for non-adherence to the recommendations included lack of awareness, preference for individual clinician experience, and concerns of missing VNT. Furthermore, the publication of the Baveno VII recommendations in 2022, which endorsed non-invasive criteria for diagnosing CSPH and initiating NSBB therapy [4], may partly explain clinician unawareness or limited uptake in earlier cases, particularly as some patients in this cohort were included from 2021.

Interestingly, 23.8% of patients in our cohort who did not meet Baveno VII criteria for screening gastroscopy were found to have VNT, representing one of the most clinically important findings of this study. Although large multicentre validation studies have consistently demonstrated a high NPV for Baveno VII criteria, this real-world observation may explain why some clinicians remain hesitant to omit screening endoscopy. Importantly, our study was not designed to challenge the validity of Baveno VII, which has been extensively validated in larger cohorts, but rather to evaluate its implementation in routine practice. The presence of VNT in a small percentage of patients reinforces the need for careful clinical judgement and ongoing efforts to refine non-invasive risk stratification strategies.

The use of Baveno VII performed less optimally in our cohort than reported in validation studies, with an observed NPV of 76.2%. This likely reflects the relatively small sample size and the real-world nature of the cohort rather than a true reduction in test performance. While the published literature demonstrates that most patients below Baveno VII thresholds are unlikely to harbour VNT, our findings highlight that false negatives can occur and should be considered when applying the criteria in clinical practice.

Medibank, a private insurance company in Australia, provides a guide of the median costs of providing a diagnostic gastroscopy according to the Medicare Benefits Schedule, which estimates to 1,820 Australian dollars (AUD) [6]. If Baveno VII were adhered to in this patient cohort, the overall potential cost savings would amount to AUD 29,120. Further utilising this across multiple centres in the state or country could lead to much more money being saved and channelled towards other areas in need of healthcare funding.

This study was not designed to comprehensively quantify all potentially avoided screening gastroscopies. Consequently, the estimated cost savings likely underestimate the broader healthcare impact of guideline-concordant practice, particularly among patients managed without endoscopy through application of Baveno VII criteria or NSBB therapy.

We are privileged in our centre to have convenient access to TE for both inpatients and outpatients. This suggests additional patient and clinician factors that have contributed to the incomplete adherence to Baveno VII. Recent studies do show that despite a high sensitivity for detecting VNT, a significant proportion of patients are misclassified [7]. Spleen stiffness measurements have been shown to be effective in ruling out VNT, and perhaps further research into combining NIT can further improve the accuracy in determining patients who can safely avoid screening endoscopies [8].

Overall, these findings support the utility of Baveno VII in reducing unnecessary endoscopic procedures but also suggest a potential role for adjunctive NIT or clinical risk stratification tools to further enhance diagnostic accuracy.

This study has several limitations. First, its retrospective, single-centre design may limit the generalisability of the findings to other healthcare settings. Second, the relatively small sample size, particularly the low number of patients meeting eligibility criteria after application of the inclusion and exclusion criteria, reduces the precision of the diagnostic performance estimates and may explain the lower NPV observed compared with larger validation studies. In addition, cost analyses were based on estimated procedural costs and did not account for downstream healthcare utilisation or long-term clinical outcomes. Future multicentre prospective studies with larger cohorts are needed to better evaluate the real-world implementation of Baveno VII, identify barriers to guideline adherence, and assess the impact of incorporating additional non-invasive markers, such as spleen stiffness measurement or other risk stratification tools, to further improve the identification of patients with VNT.

Further research is essential to refine risk stratification strategies and ensure that patients with CSPH or VNT are accurately identified without overutilising endoscopy. While current NIT like TE and platelet count are valuable, there remains a small but clinically important proportion of patients who may harbour VNT despite meeting Baveno VII exclusion criteria. To address this, ongoing studies are investigating the addition of complementary NIT, such as spleen stiffness measurement, to enhance diagnostic accuracy. Ultimately, improving adherence to Baveno VII and evolving it with robust validation could provide a safer and more efficient framework for managing cirrhotic patients, ensuring that high-risk individuals receive timely intervention while minimising harm in low-risk cohorts.

## Conclusions

This study highlights the challenges and opportunities associated with implementing Baveno VII recommendations in routine clinical practice. While large validation studies have demonstrated the high NPV of Baveno VII criteria for excluding VNT, our real-world cohort identified a small but clinically important number of patients with VNT who would not have met criteria for screening endoscopy. These findings support the use of Baveno VII as an effective strategy to reduce unnecessary invasive procedures, minimise patient burden, and generate healthcare cost savings. At the same time, they underscore the importance of ongoing clinical judgement and further refinement of non-invasive risk stratification tools to improve identification of patients who remain at risk of VNT despite meeting low-risk criteria.
